# An Evaluation of Medication Adherence in New Tuberculosis Cases in Ankara: A Prospective Cohort Study

**DOI:** 10.3390/healthcare12232353

**Published:** 2024-11-25

**Authors:** Sahin Can Ozaltun, Levent Akin

**Affiliations:** Department of Public Health, Faculty of Medicine, Hacettepe University, Ankara 06230, Türkiye; leventakin@baskent.edu.tr

**Keywords:** TB, tuberculosis treatment, medication adherence, prospective cohort studies, generalised estimating equations

## Abstract

Background/Objectives: The objective of this study was to investigate the factors influencing adherence with tuberculosis medication therapy. Non-adherence can result in treatment failure, ongoing infectiousness, and the development of drug resistance. Therefore, understanding the reasons behind non-adherence is crucial for achieving the World Health Organization’s target of 90% treatment success. Methods: Data were collected prospectively from a cohort at three different tuberculosis dispensaries (TBDs), with participants being followed up with at face-to-face visits every two months for a total of three visits. Results: In this study, the adherence rates among participants were the highest during the intensive treatment phase (81.0% at the first follow-up) but declined during the continuation phase, reaching 69.4% at the second follow-up (at the end of the fourth month of treatment) and 71.1% at the third follow-up (at the end of the sixth month of treatment) according to self-reports for the past 30 days. According to the generalised estimating equations method, factors significantly associated with better adherence included knowledge of infectiousness, daily access to medication, workplace permissions, high household income, regular sleep patterns, extrapulmonary TB, secondary education, and no alcohol consumption. Conclusions: Non-adherence with anti-TB medication has been observed in patients with TB, particularly during the continuation phase of treatment. Interventions targeting patients who experience frequent forgetfulness, adverse drug reactions, or a lack of workplace flexibility may help to improve adherence. In addition, providing personalised health education that highlights the risks of non-adherence and emphasises the infectious nature of TB could improve understanding and commitment to treatment. Ensuring regular follow-ups and support, particularly for those with lower socioeconomic status or limited social support, can further reinforce the importance of adherence in TB treatment outcomes.

## 1. Introduction

Tuberculosis (TB) has historically been one of the most lethal infectious agents. However, with the advent of effective treatment regimens, it is no longer one of the top ten causes of death as of 2019. Nevertheless, TB remains a significant global health problem with an estimated 10.8 million new cases and 1.25 million deaths in 2023 [1]. The World Health Organization (WHO) launched the End TB Strategy in 2014 to tackle this persistent problem. The indicators of this strategy are a 90% reduction in the incidence of TB and a 95% reduction in the TB mortality rate by 2035. To achieve this, a comprehensive public health approach coupled with effective diagnosis and patient-centred care will be needed [2].

According to 2018 data, our country achieved a treatment success rate of 84.3% for new TB cases, 74.4% for multidrug-resistant TB (MDR-TB), and 70.0% for previously treated cases, reflecting the ongoing challenges in TB control [3]. Ensuring that every new patient with TB adheres to the full course of TB medication through directly observed therapy (DOT) is essential to prevent the emergence of MDR-TB and reduce relapse rates [4]. The literature consistently shows that adherence is critical for favourable treatment outcomes, while non-adherence leads to undesired clinical outcomes and represents a significant barrier to global TB elimination efforts [5]. In particular, irregular medication use can lead to higher bacterial loads, an increased risk of drug resistance, and prolonged infectiousness [6,7]. In addition, mathematical models indicate that improving adherence by reducing missed doses among patients with TB could potentially lower TB incidence by up to 12% [8].

Behavioural change is a critical factor to consider in improving medication adherence. An accurate measurement of adherence is essential as it is a direct indicator of the effectiveness of a health intervention [6]. There is no gold standard method for measuring adherence. However, both process-oriented and outcome-oriented approaches are commonly used. Adherence can be measured dynamically by attending appointments on time, through questionnaires, or through pill counts and can also be estimated based on treatment success [9,10]. However, the lack of a standardised definition of TB adherence poses a challenge for the measurement and comparability of results [6]. The World Health Organization classifies the determinants of adherence into five categories: economic and structural factors, patient-related factors, the complexity of the treatment regimen, supportive communication between healthcare providers and patients, and the model of healthcare delivery [10]. Studies investigating factors influencing adherence have shown that adherence often decreases during the continuation phase of treatment [11,12].

Adherence behaviour is influenced not only by the individual patient but also by social and environmental factors and requires systematic approaches to behaviour change. Therefore, patient psychology should be considered as an important determinant of medication adherence [6]. The COM-B model is a behavioural theory that can be applied to improve medication adherence. The COM-B model, which was developed to address this need, posits that behaviour is shaped through the interaction of three factors: capability, opportunity, and motivation [13].

Capability refers to the psychological and physical skills required for adherence; opportunity refers to the environmental and social conditions that make the behaviour possible; and motivation refers to the individual’s desire or automatic drive to engage in the behaviour. Adherence to TB treatment is related to all components of this model and is influenced by factors such as the complexity of the treatment process, a lack of knowledge about the disease, and social stigma [13].

The World Health Organization has identified adherence to anti-TB medications as a multifaceted and complex problem that requires special attention through the provision of person-centred care. Therefore, patient-centred care based on patients’ values and needs should be implemented, and adherence should be promoted with counselling and social support; additionally, directly observed treatment should be implemented when necessary [14,15].

In TB treatment, the overall level (i.e., the proportion of missed doses), the timing (i.e., whether non-adherence occurs at the beginning of treatment, consistently throughout the treatment period, or mainly towards the end of treatment), and the intensity and pattern of non-adherence (i.e., whether several consecutive doses are missed or whether the missed doses are spread relatively evenly over the treatment period) are critical aspects that influence treatment outcomes and the risk of drug resistance. It is important not only to measure adherence at a single point in time but also to monitor these patterns continuously over the long course of treatment in order to capture the full extent of adherence behaviour. Studies have shown that adherence, particularly during the intensive phase of treatment when bacterial loads are the highest, is crucial for preventing early treatment failure and reducing the risk of transmission. However, adherence tends to decline over time, often more so during the continuation phase, when patients may perceive themselves to be improving [16].

The global expansion of digital technology and Internet access provides a significant opportunity to address the challenges of TB management. Video-Observed Therapy (VOT) offers a preferable solution compared to DOT by maintaining patient privacy and reducing the need for clinic visits. This approach allows for the early identification of factors that may contribute to non-adherence, and individuals with a high likelihood of non-adherence can be prioritised for VOT monitoring from the intensive assessment [6]. Analysing adherence patterns and tailoring interventions accordingly can significantly improve TB treatment outcomes. Enhanced digital monitoring approaches, such as Video-Observed Therapy (VOT), have shown promise in identifying patterns of non-adherence in real time, which could lead to more targeted and effective interventions [16].

Research on the factors contributing to non-adherence in Turkey is limited [17,18,19]. To elucidate the dynamic and complex nature of adherence, this study aims to assess the level of adherence at different stages of TB treatment and identify factors influencing adherence using repeated measures in a prospective cohort study.

## 2. Materials and Methods

**Figure 1:**
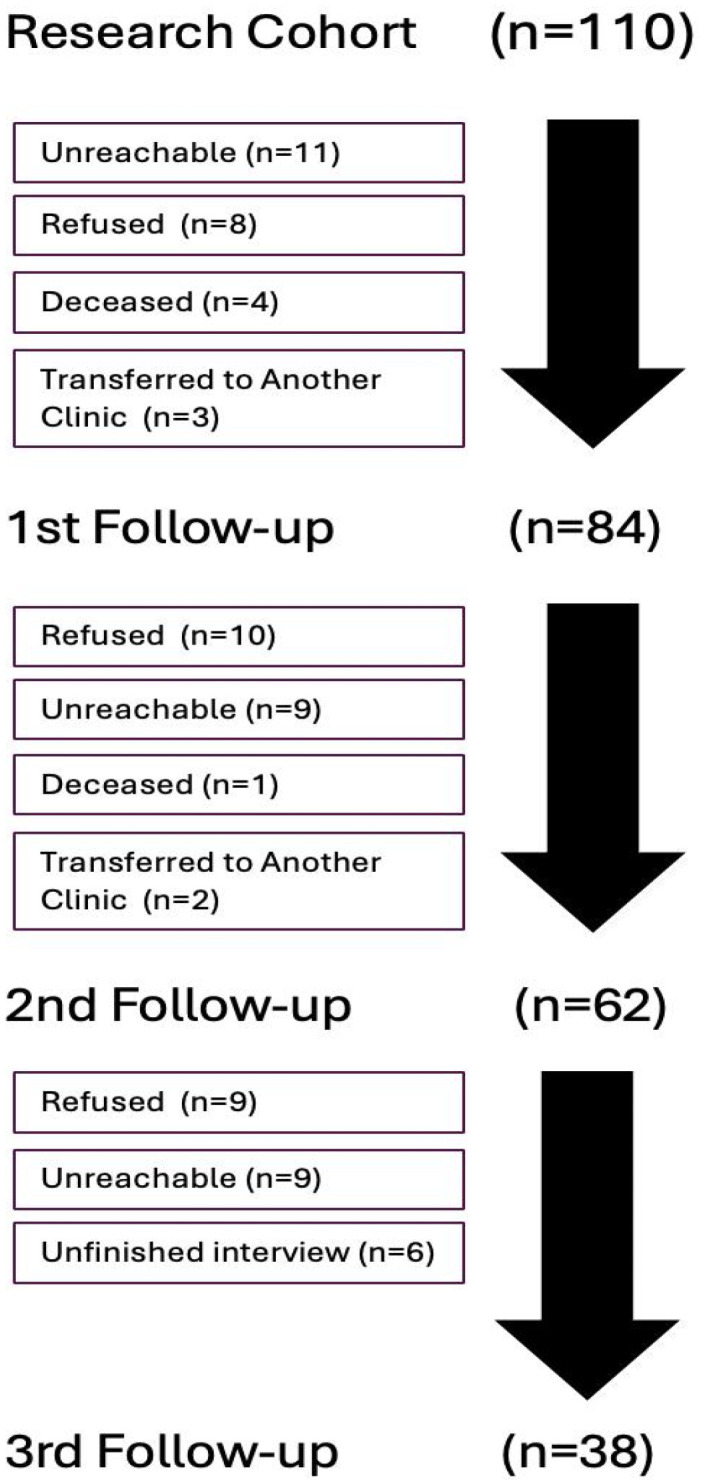
A flowchart of the study.

The study cohort consisted of individuals who were diagnosed with TB between 1 October 2018 and 1 January 2019. Due to the study design, patients were followed up with for 6 months. As the last case was diagnosed on 1 January 2019, the follow-up period ended on 30 June 2019. The researcher invited patients to participate in the study at the time they presented to the TBD for treatment. The 110 patients who attended the clinic were informed about the study, and of these, 84 agreed to participate, signed the informed consent form, and were enrolled in the study for the first follow-up. Of the 110 patients who were informed about the study, 8 refused to participate. Reasons for refusal included a lack of time to attend follow-up visits and a general unwillingness to participate in the study. There were no statistically significant differences in age, gender, and case type between the 110 patients screened and the 84 who agreed to attend the first follow-up (Figure 1).

The three TBDs selected were chosen for their stable and experienced staff and reliable patient registration practises, which increase the reliability of the data. In addition, these TBDs cover both low and high socioeconomic areas of the city, providing a more comprehensive representation. The TB cases diagnosed in five TBDs in Ankara had similar sociodemographic characteristics (such as age distribution and female predominance) and case characteristics (mainly extrapulmonary TB) to the 84 patients included in the first follow-up of the study [20].

### 2.1. Ethical Approval and Informed Consent

This study was conducted in accordance with the Declaration of Helsinki and was approved by the Hacettepe University Non-Interventional Research Ethics Board (decision date: 14 September 2018; decision number: 18/896-05). Prior to the interviews, participants were provided with both verbal and written information about the purpose and structure of the interviews, and they signed an informed consent form to participate. For participants under the age of 18, permission was obtained from both the participant and their parents before the participants were asked to sign the informed consent form. The data collection phase of the study was approved by the Ankara Provincial Health Directorate (decision date: 25 October 2018; decision number: 62693856-604.02).

### 2.2. Data Collection and Follow-Up

Standard TB treatment consists of a two-month intensive phase with four antibiotics, followed by a four-month continuation phase with two antibiotics. The first day of treatment was considered day 1, and subsequent follow-up points were scheduled accordingly. At the end of the two-month intensive treatment phase (between days 40 and 60), patients completed the first data collection form. Patients were then interviewed prospectively at two additional follow-up points: between days 100 and 120 (second follow-up) and between days 160 and 180 (third follow-up).

During the three periods of follow-up periods, data were collected by a single interviewer using a structured questionnaire, which was administered face-to-face by the researcher after the patients had received health services at the TBD. Of the 84 patients who initially participated in the study, some patients could not be followed up with at the second and/or third follow-up. Reasons for missed follow-up(s) included patients not attending the health facility on the scheduled day, arriving earlier than the scheduled day without informing the researchers, a lack of availability due to work commitments, and being out of town during the follow-up period.

In order to develop the data collection form, a comprehensive review of the literature was conducted to identify factors contributing to non-adherence to TB treatment [21,22,23,24]. The results show that only one scale specifically designed to measure adherence to TB treatment was available [21], but its Turkish validity and reliability had not been established. In the second phase, routine information collected during the registration of new patients at the TBD [25] and details provided in the TB Patient Information Guide issued by the Ministry of Health [26] were reviewed and supplemented with expert advice from TBD physicians.

Based on the findings and recommendations, a data collection tool was developed in the third phase. This tool included data from medical records for only four items—diagnosis, case type, treatment start date, and initial symptoms. The remaining questions were directly addressed with the patient. In the fourth phase, a pilot test was conducted with five patients with TB receiving TB treatment at another TBD in Ankara, outside of the three designated study sites. After necessary adjustments, an 83-item data collection form was administered by a researcher in the first follow-up phase of the study.

The independent variables cover a range of characteristics, including socioeconomic (e.g., level of education, health insurance, and monthly income), health system-related (e.g., place and frequency of obtaining medications and the need to take time off work for treatment), patient-related (e.g., gender, age, place of birth, presence of chronic diseases, smoking, alcohol consumption, and regular sleeping habits), disease-related (e.g., presentation of symptoms at diagnosis, emotional response to learning of TB diagnosis, presence of drug resistance, and case type), and treatment-related (e.g., knowledge of risks associated with irregular medication use or feeling bad when obtaining medication) characteristics.

Certain variables (date of birth, place of birth, gender, marital status, level of education, occupation, health insurance status, type of housing, household size, emotional response to learning of the TB diagnosis, drug resistance status, and the level of information provided by the physician about medication use and potential side effects) collected from the medical records [25] were excluded from the administration of the second and third follow-ups, resulting in a 70-item data collection form for these follow-ups.

### 2.3. Variables

The dependent variables of this study were defined as “full adherence with medication treatment” and “non-adherence with medication treatment”. In some studies, instead of assessing the number of missed doses in the previous month [23], researchers assessed medication adherence over the previous seven days [21,27]. The assessment of medication adherence with medication was based on the number of doses missed in the previous 30 days, as reported in an open-ended format. Days on which patients did not take their medication on the advice of their physician because of adverse effects were not counted as days of non-adherence with medication therapy. Self-reports are subject to recall and social desirability bias. To minimise these biases and to verify whether there were any doctor-recommended medication breaks due to side effects, the patient’s follow-up physician was interviewed at the TBD follow-up visit and the information was cross-checked.

Participants were asked by the interviewer to answer the questions on the data collection form based on their circumstances in the past 30 days (e.g., the need to take time off work for medical appointments, monthly income, regular sleep patterns, feeling unwell when taking medication, etc.).

As in the literature [23,28,29], the term “full adherence” is defined as taking all prescribed medications, whereas “non-adherence” is defined as missing at least one daily dose of TB medications within the past 4 or 30 days. The duration variable is defined in terms of the dates of the follow-up processes. Those who reported consuming alcohol at least once over the previous 30 days were classified as alcohol consumers.

In the univariate analyses and the generation of the model through the GEE method, certain classifications were employed. Age and gender variables were converted into categorical data in order to make comparisons with the literature. Participants were divided into four age categories: those aged 0–18 years, 19–49 years, 50–64 years, and those aged 65 years and older. For univariate analysis, data on household income per capita were grouped according to the median value, which was set at EUR 6.35. The variables “regular sleep pattern” and “feeling unwell when obtaining medications” were dichotomised based on responses to a five-point Likert scale.

Patients can obtain their medications from a variety of sources, including Family Health Centres (FHCs), TB hospitals, or TBDs. Therefore, the places where medications are obtained are grouped as TBDs and TB hospitals. Responses regarding the frequency of obtaining medications were categorised as daily, every 7–14 days, and monthly. Problems with obtaining permission from their workplaces to obtain medications and those who did not experience such problems were treated as separate variables, while non-working patients or those who did not need to obtain permission were included in the same classification.

### 2.4. Statistical Analysis

The data analysis was conducted using the IBM SPSS version 23.0 software. A 5% significance level was established for the purposes of statistical significance (*p* < 0.05). The evaluation of differences between groups was conducted using the Pearson chi-square test and Fisher’s exact test when necessary. Repeated measures data from clinical trials designed as “longitudinal studies” were analysed using mixed-effects models and GEE methods. In this study, a non-parametric GEE method was chosen to perform longitudinal analyses on categorical data. The GEE method was used to examine the factors influencing medication adherence, taking into account the effect of time variables. The time variable was always included in univariate analyses using the GEE method. Results are presented in tabular form, including the corresponding odds ratios, 95% confidence intervals, and *p* values.

Data from patients aged 0–5 years (n = 4) were included only in the descriptive tables (n = 84) as their responses, provided by their parents, could have introduced bias into the model standardisation. Therefore, these data were excluded from both univariate and multivariate analyses. As the aim was to capture the perceptions of adherence of patients actually receiving treatment, the inclusion of this age group could have introduced bias into model. Therefore, the GEE method was performed using data from the 80 cohort participants who responded to at the first follow-up, as this age group is unlikely to have an understanding of a lengthy treatment process and its challenges that is critical for assessing adherence-related factors. However, two children aged 14 and 15 were included as they were considered to have sufficient understanding of the disease and treatment to respond meaningfully to the adherence-related questions.

The fact that the GEE method with backward elimination was used in the analysis supports the validity and reliability assessment of the data collection form. In addition, the fact that no high-level correlations were identified in the correlation matrix indicates that there was no problem of multicollinearity in the data set and that the variables contributed to the model independently.

In order to obtain a model comprising the fewest possible variables that significantly affect medication compliance (*p* < 0.05), a backward elimination method was applied using a GEE analysis. Only variables with a Type I error rate lower than 20% were included in the model. In addition, age groups and gender were included in the model as potential confounding variables, as they have been identified as risk factors for non-adherence in numerous studies [30].

Prior to this, the variable indicating whether patients felt unwell when obtaining medications, which demonstrated an effect on medication compliance (*p* = 0.032), lost statistical significance (*p* = 0.082). However, it was retained in the model as it provides insight into the mental health conditions of the patients. This variable was included alongside other internationally accepted confounding variables to ensure a parsimonious yet comprehensive model. The retention of this psychological factor was intended to demonstrate that the significant variables remained effective even when this element was controlled for.

## 3. Results

Among the patients involved in our study, 52.4% (n = 44) were female, with a mean age of 44.74 ± 19.7 years and an age range spanning from 6 months to 84 years. A total of 92.9% (n = 78) participants were born in Turkey. Data on educational level, health insurance, and monthly income are presented in Table 1. 

Among the patients who completed their first follow-up, pre-diagnosis symptoms were documented as fatigue in 41.7% (n = 35), cough in 31.0% (n = 26), and weight loss in 31.0% (n = 26). A total of 65.5% (n = 55) of the participants reported the presence of chronic diseases. The most common chronic diseases among these individuals were hypertension (30.9%), diabetes mellitus (16.7%), and asthma (12.7%). Extrapulmonary TB was found in 52.4% (n = 44) of the individuals, while 6% (n = 5) had both pulmonary and extrapulmonary TB. Upon receiving their TB diagnosis, 33.7% of patients reported feelings of sadness, 32.9% were neutral, and 30.1% felt fear. The incidence of resistance to any TB medication during treatment was 8.4% (n = 7) (Table 2).

**Figure 2:**
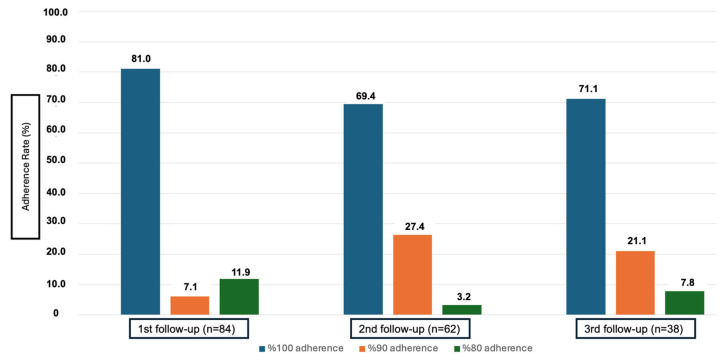
Adherence rates in tuberculosis treatment according to follow-up phases (Ankara, 2019).

The proportion of individuals who were fully adherent was 81.0% at the first follow-up, 69.4% at the second follow-up, and 71.1% at the third follow-up (Figure 2).

**Figure 3:**
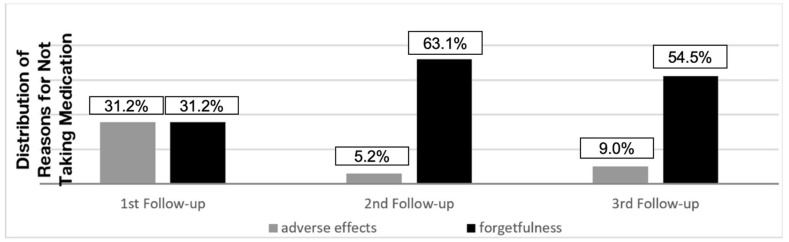
Distribution of reasons for not taking TB medication according to follow-up reports in study cohort (Ankara, 2019).

The frequency of not taking medication due to adverse effects was found to be 31.2% in the first follow-up, 5.2% in the second follow-up, and 9.0% in the third follow-up. The frequency of not taking medication due to forgetfulness was found to be 31.2% in the first follow-up, 63.1% in the second follow-up, and 54.5% in the third follow-up (Figure 3).

No statistically significant association was found between complete adherence to medication therapy and factors such as gender, age, place of birth, educational level, health insurance coverage, and family income per capita (*p* > 0.05). A multivariate analysis using the GEE model revealed variables that significantly influenced full adherence to medication therapy (Table 3). Participants who were aware that consistent medication use would inhibit transmission were 8.18 times more likely to adhere to medication therapy than those who were not aware (%95 CI = 2.67–25.04; *p* < 0.001). Participants who received daily medication were 2.66 times more likely to be fully adherent than those who received monthly medication (95% CI = 1.14–6.18; *p* = 0.02).

Participants who obtained their medication from the TBD or TB hospital were 6.9 times more likely to be fully adherent than those who obtained it from the Family Health Centre (95% CI = 1.85–26.26; *p* = 0.004). Individuals who experienced no difficulties in obtaining time off work to obtain their medication were 10.5 times more likely to be fully adherent than those who experienced difficulties in obtaining time off work (%95 CI = 1.24–89.24; *p* = 0.03). Individuals with a higher per capita household income were 7.4 times more likely to be fully adherent than those with a lower income (%95 CI = 2.32–23.60; *p* = 0.001). Individuals who typically maintained a regular sleep pattern were 3.2 times more likely to be fully adherent to medication therapy than those who rarely slept regularly (%95 CI = 1.09–9.38; *p* = 0.03).

Patients with extrapulmonary TB were four times more likely to be fully adherent to medication therapy compared with those with pulmonary TB (%95 CI = 1.14–14.14; *p* = 0.03). Individuals with at least a secondary education had an 8.5-fold increased likelihood of complete adherence to medication therapy compared to those with less education (%95 CI = 2.03–36.04; *p* = 0.003). Non-alcohol users had a 35-fold increased likelihood of complete adherence to medication compared with alcohol consumers (%95 CI = 3.72–329.61; *p* = 0.002).

## 4. Discussion

### 4.1. Frequency of Adherence to Drug Therapy in Patients

It was observed that full adherence to TB treatment decreased after the transition to the continuation phase of treatment (Figure 2). The decrease in motivation and the weakening of the perception of the importance of treatment as time progresses may have contributed to this [11]. When the continuation phase of treatment is started towards the end of the second month, most complaints disappear. Therefore, while adherence with treatment decreases in this period [23], the frequency of treatment non-adherence is higher in the maintenance period of treatment compared to the initial period [22]. This perception may lead patients to perceive the treatment as less necessary, which may decrease adherence to treatment.

Additionally, an increase in the 90% adherence rate was observed in the second and third follow-ups, indicating that some patients maintained adherence with irregular medication intake. In another study conducted on TBD in Turkey, similar to ours, where adherence was measured using a more sensitive and cross-sectional study design, similar adherence levels were observed [20]. This finding is consistent with other studies showing a decrease in adherence when patients enter the continuation phase [11,12].

As the follow-up period progressed, it was observed that the number of patients above 90% adherence increased, but there was a decrease in the frequency of those with full adherence. Other studies also classify and evaluate adherence in this manner [31,32]. Drug side effects that cause a decrease in patient adherence are generally observed in the first three months of treatment [33,34]. In the present study, drug side effects were the most common reason for not taking medication at the first follow-up (31.2%) (Figure 3). In the follow-ups after switching to maintenance treatment, more than half of the patients who did not take medication stated forgetfulness as the reason.

According to the patients’ reports, the most common reason for non-adherence in all follow-ups was identified as forgetfulness, as also reported in the literature [23,35]. People with forgetfulness ought to request help from family and community members to maintain their treatment plan, which comprises the application of DOT. Social support from family, the community, and organisations plays a vital role in TB treatment adherence. This support helps patients to adhere to treatment by reducing their stress and increasing their self-efficacy. For patients with forgetfulness, active provision of family and community support by healthcare professionals may increase the adherence of these patients with DOT-based treatment [36,37].

At the beginning of treatment, the most common side effects that may occur with the drugs they use should be explained to the patients. Side effect questioning should also be performed during daily medication intake in patients who receive TBD. Since forgetting to take medication is a risk that increases as the treatment continues, reminder SMS messages can be sent to patients who cannot be followed up with during the treatment period. In addition, it is also important to follow up on missed appointments.

In this study, it was anticipated that participants might overestimate their adherence for various reasons (e.g., interviewer bias, recall bias, social desirability bias, and volunteer bias), although the number of days without medication was verified with their doctors. Therefore, in the analyses, only those who were confirmed to have taken all of their medication completely (100% adherence) were considered compliant. In order to identify the reasons for non-adherence more precisely in this way, not taking even one medication was considered as non-adherence.

The adherence measurements in this study relied on self-reports, which are susceptible to recall bias and social desirability bias. Furthermore, adherence may have been overestimated due to volunteer bias, as individuals willing to participate in the study could have an inclination towards adherence. Using a single interviewer for all participants may have influenced them to report adherence more favourably, thus possibly inflating adherence rates. The adherence rate was calculated based on participants who completed the study. Considering the effect of volunteer bias, adherence might have been lower if the entire cohort had attended all follow-ups [38].

To control for attrition bias, we compared patients who completed all three follow-ups with those who missed the second or third follow-up. No statistically significant differences were found between these groups in terms of gender, age, marital status, education, or income (*p* > 0.05), indicating limited attrition bias. These factors collectively suggest that adherence levels may have been overestimated.

### 4.2. Factors Affecting Adherence According to GEE Method

In the following paragraphs, the factors associated with non-adherence identified through the GEE method will be discussed.

Individuals with at least secondary-level education were statistically significantly more likely to achieve full adherence. Two studies in Turkey found a positive relationship between education level and adherence, though this association was not statistically significant [18,19,20]. In a study conducted in Brazil, it was found to be statistically significant that adherence was higher in individuals with at least 8 years of education and in individuals with at least 6 years of education in a study conducted in South Africa [39,40]. Higher-educated people may have better health literacy, which would help them to understand the need for following their treatment plans [36].

Higher per capita family income was statistically significantly associated with a higher likelihood of achieving full adherence. Studies have demonstrated that lower income [41] and income lower than minimum wage [42] are associated with non-adherence in medication use. A study conducted in a TBD in Turkey found that patients with poor economic conditions were significantly more likely to miss their medication during treatment [20].

Economic difficulties, combined with a lack of social support and substandard living conditions, may impede individuals’ ability to adhere to treatment programmes. Although the Turkish government provides free diagnosis and treatment for TB, low-income patients may still struggle to afford essential needs, such as food and transportation, during their treatment. Consequently, it is vital to sustain and expand the financial aid programme, initiated in 2017, to support patients with TB, including those who are foreign nationals.

The healthcare facilities where patients obtained their medications and the frequency of medication procurement were also influential on adherence. Patients who accessed their medication at specialised TB facilities or received it on a daily basis showed higher rates of adherence. A poor relationship between the patient and health worker and a lack of health education are important determinants of non-adherence [43].

Patients obtaining medications from hospitals or TBDs had better adherence than those obtaining them from family health clinics (FHCs). This difference in adherence could be explained by the increased monitoring of these establishments by experts on TB. In primary healthcare organisations, TBDs provide more accurate patient-oriented communication, and the treatment process and side effects are managed in a way that does not cause incompatibility.

The fact that people who visited health institutions to obtain medicines every day or who received inpatient treatment in the hospital were in constant contact with health professionals may have increased their medication adherence. DOT should be planned specifically for each patient. The most appropriate place and time should be determined for the patient, taking into account their place of residence and working status [4].

The main factors that make it difficult for patients to access medication and examinations include the remoteness of diagnostic and treatment centres [22], transport difficulties [44], unemployment [44] and workplace leave problems [45]. Studies show that almost a third of patients had problems taking their medication regularly and on time, and more than a third of patients had to take time off work to collect their anti-TB medication [46].

Increasing the number of dispensaries is important for patients to have easier access to treatment; this may increase patients’ adherence rates, especially by reducing transport time and costs. In this study, it was observed that patients who had to take time off from work to obtain medication had lower adherence rates. Considering the effects of a low income level and difficulties in taking leave at work on non-adherence, it is necessary to develop financial and social support mechanisms for both employed and unemployed patients. Providing these supports may reduce barriers in the treatment process and promote patient adherence. In order to increase patient adherence, flexible working hours can be implemented in dispensaries. Flexible working hours in dispensaries can be considered as another measure to increase patient adherence. In addition, coordination between the patient’s workplace manager and the TBD physician, while respecting patient confidentiality, may facilitate support for patients during workplace leave processes and make their access to treatment sustainable.

In this study, it was observed that the frequency of treatment adherence was higher in extrapulmonary tuberculosis cases. Patients with extrapulmonary tuberculosis showed higher rates of compliance, which is likely attributable to the absence of stigma or anxiety associated with transmission. A study conducted in Turkey reported no significant difference in adherence between pulmonary and extrapulmonary TB cases [18]. In another study conducted in Turkey, it was reported that pleural tuberculosis cases were found to be more compliant compared to pulmonary tuberculosis cases, whereas the lowest adherence rate was observed in other extrapulmonary tuberculosis cases [19]. In addition, in the same study, the absence of haemoptysis and the radiological absence of cavitary appearance were also found to be associated with treatment adherence [19]. In this study, the adherence of patients without haemoptysis in the first follow-up was observed to be higher, but no significant difference was found.

The need for invasive approaches to diagnose extrapulmonary tuberculosis cases may have led these patients to attribute more importance to treatment than patients diagnosed by sputum examination alone. Since both patient groups were treated with the same drugs, the difference in the diagnostic process may have an effect on adherence. However, the presence of only two cases of pleural tuberculosis in our study limited us to evaluate the difference between extrapulmonary tuberculosis cases and pleural tuberculosis cases. The retrospective nature of both studies, the low number of cases, and the level of participation in the present study emphasise the need for more comprehensive studies to evaluate such relationships in more detail.

The primary reason for the stigmatisation experienced by patients with pulmonary TB is their infectiousness [47]. Besides stigma, the social isolation experienced by patients with pulmonary TB due to being infectious for at least three weeks, coupled with the economic burden, may also reduce adherence. Our study supports an association, as it was observed that treatment adherence is higher among non-infectious extrapulmonary TB cases. In this context, it is an understandable finding that patients who knew that taking regular medication would end infectiousness were more compliant. However, several studies have shown that the specific organ affected by tuberculosis has a negligible effect on treatment adherence [47]. Those who were aware that transmission could continue in the absence of regular medication intake showed higher levels of medication adherence, possibly due to the need to maintain employment or participate in social activities. Previous research has shown that patients’ awareness of the advantages of treatment positively influences treatment adherence.

TB education and support programmes provided to healthcare workers, patients with TB, and communities at risk of TB could potentially reduce the stigma associated with the disease. To improve adherence, health education and counselling on TB and its treatment should be provided at the outset and throughout the treatment process [35,36]. Patients who were knowledgeable about the disease and its treatment showed higher levels of adherence [48].

Health-promoting behaviours, such as maintaining a regular sleep pattern and avoiding alcohol, positively influenced adherence. Alcohol consumption, similar to TB medications, carries a risk of hepatotoxicity. As a result, alcohol-induced hepatotoxicity may have temporarily limited the use of these medications, possibly contributing to decreased patient adherence.

A retrospective study in Turkey showed no difference between alcohol consumers and non-consumers regarding non-adherence [13]. However, patients who were not drinkers or light drinkers showed significantly higher treatment success compared to moderate or heavy drinkers [42]. When the effect of the 10-question alcohol use disorder test and the level of alcohol consumption developed by the WHO on adherence was analysed, moderate- and high-risk alcohol consumption were found to be associated with non-adherence in another study [49].

These findings suggest that the effect of alcohol consumption on adherence was related with the level of consumption. At the beginning of treatment, the level of situations that may create a risk of non-adherence, such as alcohol consumption, should be questioned in detail.

A low income and regular alcohol consumption may facilitate the emergence of risky health behaviour, such as non-adherence. Alcohol misuse may have reduced medication adherence by causing hepatotoxicity or impaired judgement [50]. Alcohol use can be considered as a criterion for assessing the adherence of patients with TB to their physicians’ health advice. This may reflect patients’ tendency to disregard health advice outside of the prescribed TB treatment programme (e.g., taking medication regularly and keeping follow-up appointments).

In a study presenting the findings from the only adherence scale [21] developed specifically for TB in the literature, the importance of a balanced diet and regular sleep was emphasised for improving adherence. Sleep disorders may affect areas such as attention, decision making, and risk-taking and may trigger non-adherence behaviours such as patients not taking their medication regularly. This emphasises the importance of sleep patterns in the treatment process [51].

### 4.3. Implications for Policy and Practice

The factors identified in this study regarding non-adherence to TB treatment can be interpreted through the COM-B model, providing insights for policy and practice. Addressing capability involves ensuring patients possess the necessary knowledge and skills to adhere to treatment [13]. Enhancing patients’ understanding of TB and its treatment process, particularly the consequences of irregular medication intake on maintaining contagiousness, could reinforce motivation to adhere. For patients with lower education levels, using clear and visually supported educational materials may improve adherence by making treatment information more accessible and understandable [24].

Environmental and social factors are critical in shaping adherence behaviours [13]. Effective DOT programmes that ensure regular interaction between patients and healthcare workers are essential for consistent monitoring and adherence. Accessibility to daily medication at specialised TB facilities staffed with experienced professionals is beneficial as these interactions provide support and minimise missed doses. Social support mechanisms, such as transportation assistance for low-income patients, are also recommended to sustain adherence over time.

Both intrinsic and extrinsic motivations are fundamental for adherence. Health-promoting behaviours, such as regular sleep, which supports overall health and motivation, should be encouraged. Patients with sleep disorders could benefit from health education and specific interventions to regulate sleep [13]. Understanding that irregular medication intake can heighten infectiousness could serve as a powerful motivational factor, helping patients recognise the public health impact of adherence and bolstering their commitment to the treatment journey. Emphasising the role of adherence in public health through education could further strengthen motivation among patients [24].

## 5. Strengths and Weaknesses

This study’s strengths include the use of the GEE method in the repeated measurement analysis to explore factors influencing adherence and its distinction as the first prospective cohort study on TB adherence in Turkey. By performing three follow-ups—once at the beginning of treatment and twice during the continuation period—rather than a single follow-up over the typical six-month medication treatment period, the study obtained more consistent and reliable data on adherence patterns [9].

Despite the limited sample size (n = 84), conducting the study across three of the five TB clinics in Ankara—a city with a population of 5.8 million— may provide a limited level of representativeness within this urban setting. Including all clinics could have yielded more comprehensive data, but it could not be determined whether the selection of clinics might have had positive or negative impacts on adherence as no significant differences in terms of gender, age, or case type (pulmonary and extrapulmonary TB) were observed between the selected and unselected clinics. This suggests that the findings may reflect adherence behaviours within Ankara’s urban context, though the limited sample size may still impact the broader applicability of the results to other populations.

Due to financial constraints, all face-to-face interviews were conducted by a single interviewer. While this approach ensured consistency in the data collection process, it may have introduced interviewer bias. Involving multiple interviewers could have minimised this risk by providing more diversified data collection.

Although this study was completed with only 33.3% of the cohort, which could introduce attrition bias, an analysis of demographic variables (age and gender) and case type showed no significant differences between those who continued in the study and those who dropped out. This suggests that the impact of attrition bias on the representativeness of the remaining sample may have been minimised, though it does not completely eliminate the possibility of bias due to the high loss to follow-up.

To minimise potential dropouts, conducting follow-ups at shorter intervals (e.g., once a month) rather than every two months in multi-centre studies may help maintain participants’ engagement and reduce dropout rates. Additionally, sending reminder messages to cohort participants before each follow-up can boost motivation and encourage continued participation in the study.

With forgetfulness being a primary reason for non-adherence, monitoring all patients through DOT is appropriate. In accordance with the protocol established in 2017 between the Ministry of Health and the Ministry of Family and Social Policies, financial assistance is provided to eligible Turkish patients with TB undergoing a designated DOT method. These methods include home DOT, clinic, or video/tele-DOT. Video/tele-DOT, which is favoured by patients due to its efficiency in saving both time and money, requires broader implementation. Given that forgetfulness was the most frequently reported reason for missing doses across all follow-ups and that adherence declines over time, implementing effective DOT strategies, such as tele-DOT, could enhance adherence by promoting regular medication use. A national software platform should be developed to notify physicians of missed doses for timely intervention. Text reminders can be sent to patients without smartphones.

As Ankara is a large city with a population of 5.8 million, there is a need to increase the number of TBDs providing primary healthcare services for TB (currently five TBDs as of 2024). Increasing the numbers of TBDs and qualified healthcare personnel in these clinics, where TB diagnosis and treatment are provided free of charge, will enhance accessibility and early diagnosis opportunities, especially for foreign patients. In areas lacking sufficient TBD, where patient access may be limited, healthcare workers at primary care centres should receive in-service training on the importance of DOT. Furthermore, the restricted availability of experienced physicians and healthcare professionals with expertise in TB constitutes a significant obstacle. Therefore, a sufficient number of health personnel working in primary healthcare organisations should be competent to manage patient treatment according to national strategies and guidelines.

## 6. Conclusions

Further prospective research is required to elucidate the factors influencing medication adherence. Understanding these factors can aid in designing healthcare interventions focused on social and psychological support to improve adherence rates. Developing a reliable and practical TB medication adherence scale for field use will enable an ongoing assessment of patient adherence and allow for the swift identification of non-adherence risks. This approach may contribute to controlling TB by reducing non-adherence and enhancing treatment outcomes.

## Figures and Tables

**Table 1:** Sociodemographic characteristics of the study cohort (Ankara, 2019).

Variables	N = 84	% of Total
Gender		
Male	40	47.6
Female	44	52.4
Age group		
0–18 years	6	7.1
19–49 years	44	52.3
50–64 years	16	19.0
65 years and older	18	21.6
Mean ± SD = 44.74 ± 19.7; median = 43.5; min–max = 1–84		
Place of birth		
Turkey	78	92.9
Other	6	7.1
Educational level		
Primary school or less	51	60.7
Secondary school or higher	33	39.3
Health insurance		
Yes	70	83.3
No	14	16.7
Average monthly income		
EUR ≤252	20	23.8
EUR 252–315	25	29.8
EUR 315–472	16	19.0
EUR ≥472	23	27.4

Mean ± SD = EUR 376.63 ± EUR 227.24, median = EUR 315; min–max = EUR 0–1071.

**Table 2:** Tuberculosis-related characteristics of the study cohort (Ankara, 2019).

Variables	N = 84	% of Total
Initial complaints upon admission *		
Fatigue	35	41.7
Cough	26	31.0
Weight loss	26	31.0
Sweating	25	29.8
Sputum production	23	27.4
Presence of chronic diseases *		
None	29	34.5
Present	55	65.5
- Hypertension	18	21.4
- Diabetes mellitus	14	16.6
- Asthma	7	8.3
Type of case		
Extrapulmonary TB	44	52.4
Pulmonary TB	25	41.6
Pulmonary and extrapulmonary TB	5	6.0
Emotional response upon learning of TB diagnosis *		
Sadness	28	33.7
Neutral reaction	27	32.9
Fear	25	30.1
Drug resistance status of the patient		
Sensitive to first-line drugs	77	91.6
Drug-resistant case **	7	8.4

* Multiple options are specified. ** signifies resistance to any TB medication. Rifampicin resistance was detected in one case.

**Table 3:** GEE model of variables affecting full adherence to medication therapy in study cohort (Ankara, 2019).

	GEE Analysis		
Variables	N = 80	%95 CI % of Total	
Knowledge that irregular medication use will prolong transmission			
Does not know	1.00		
Knows	8.18	2.67–25.04	<0.001
Frequency of obtaining medication			
Monthly	1.00		
Daily	12.71	2.33–69.21	0.003
7–14 days	0.28	0.08–0.97	0.045
Feeling bad when obtaining medication			
Yes	1.00		
No	3.46	0.85–14.06	0.08
Place of obtaining medication			
Primary healthcare centre	1.00		
TBD/TB hospital	6.98	1.85–26.26	0.004
Taking leave from work to obtain medication			
No leave/not working	1.00		
No issues with taking leave	5.48	0.98–30.61	0.05
Issues with taking leave	0.07	0.01–0.63	0.01
Household income per capita			
EUR 92 and less	1.00		
EUR 93 and above	7.41	2.32–23.60	0.001
Sleeping regularly			
Never/rarely	1.00		
Sometimes	0.43	0.07–2.72	0.37
Mostly/always	3.21	1.09–9.38	0.03
Case type			
Pulmonary TB	1.00		
Extrapulmonary TB	4.02	1.14–14.14	0.03
Educational level			
Primary school graduate or lower	1.00		
Secondary school graduate or higher	8.55	2.03–36.04	0.003
Alcohol consumption			
Yes	1.00		
No	35.02	3.72–329.61	0.002
Gender			
Female	1.00		
Male	2.00	0.45–8.77	0.35
Age group			
0–18	1.00		
19–49	0.13	0.01–1.59	0.11
50–64	0.10	0.00–1.21	0.07
65 and older	0.28	0.02–3.63	0.33

## Data Availability

Additional data are available from the corresponding author upon reasonable request.
